# The coping strategies employed by individuals with chronic low back pain: secondary qualitative analysis of data from diverse adult populations in two sub-Saharan African countries

**DOI:** 10.3389/fresc.2024.1442789

**Published:** 2024-11-20

**Authors:** Chinonso Nwamaka Igwesi-Chidobe, Loveness A. Nkhata, Benjamin Ozumba

**Affiliations:** ^1^ School of Allied Health Professions and Midwifery, Faculty of Health Studies, University of Bradford, Bradford, United Kingdom; ^2^ Global Population Health (GPH) Research Group, University of Nigeria, Nsukka, Nigeria; ^3^ Division of Physiotherapy, Department of Health and Rehabilitation Sciences, Faculty of Medicine and Health Sciences, Stellenbosch University, Cape Town, South Africa; ^4^ Department of Physiotherapy, School of Health Sciences, University of Zambia, Lusaka, Zambia; ^5^ Faculty of Medicine, University of Nigeria, Ituku Ozalla, Nigeria

**Keywords:** chronic low back pain, coping strategies, secondary qualitative study, adaptive, maladaptive

## Abstract

**Introduction:**

The use and influence of coping mechanisms vary across cultures and evaluation methods. Limited research exists on coping mechanisms for non-specific chronic low back pain (CLBP) in African societies. This secondary qualitative study explored adaptive and maladaptive coping strategies used by adults with CLBP in Nigerian and Zambian communities.

**Methods:**

Primary qualitative data from studies in rural Nigeria and peri-urban Zambia were used. Primary data were collected through in-depth face-to-face interviews with adults living with back pain to explore their experiences and coping strategies. Thematic analyses of interview transcripts from both studies were conducted using the framework approach, combining inductive and deductive analysis techniques.

**Findings:**

Participants used a mix of adaptive and maladaptive coping strategies for CLBP. Some strategies had both positive and negative aspects, with varying effects on individuals. A few participants focused on adaptive coping, staying positive, and actively managing their pain through stretching, exercise, pacing activities, spirituality, and belief in their strategies. They avoided seeking treatment for their back pain.

**Discussion:**

The nuances in coping with CLBP should be taken into consideration when developing coping assessment strategies and rehabilitation interventions for individuals with CLBP.

## 1 Introduction

Chronic low back pain (CLBP), the majority of which is non-specific, is the cause of most of the pain and disability associated with back pain and is one of the most expensive and difficult conditions to manage globally (1, 2). The burden and impact of CLBP are overwhelming in low- and middle-income countries (LMICs), particularly in African countries (3). This might be due to high levels of poverty, lack of social security, dangerous living and working conditions, unhelpful back pain beliefs and coping strategies, and limited access to health information and resources (4–8).

Treatment for CLBP typically involves a multidisciplinary approach, including pharmacological therapy, interventional techniques, and therapies including physiotherapy (9). The effects of rehabilitation may be improved by the addition of supplements such as alpha lipoic acid, acetyl-L-carnitine, resveratrol, and cholecalciferol (10). Prolonged use of pharmacological therapies is not recommended due to their limited effectiveness and potential side effects and adverse events, therefore non-pharmacological management is the primary approach (11, 12).

Coping refers to the deliberate and conscious efforts made by individuals to effectively deal with the challenges and demands they face, whether they originate from external sources or internal factors. Coping strategies play a crucial role in mitigating the impact of pain intensity on disability and overall quality of life among individuals living with non-specific CLBP (13, 14).

Passive coping strategies, which involve withdrawing or relinquishing control to an external entity, have been associated with the progression of acute low back pain to CLBP. In addition, they have been linked to increased pain intensity, emotional distress, reduced function and work-related disability, and fear of movement (13, 15–19). Examples of passive coping strategies identified in studies include relying on others for daily tasks, perceiving an inability to control pain, hoping for improved pain medication from healthcare professionals, engaging in distractions, praying and hoping, ignoring pain sensations, using coping self-statements, and experiencing feelings of helplessness and hopelessness (15–18, 20). However, it is worth noting that certain passive coping strategies, such as praying and hoping, as well as diverting attention, have been found to be occasionally beneficial in improving outcomes related to CLBP, such as reducing pain intensity (21, 22). However, passive coping strategies such as focusing excessively on pain, limiting social activities, and relying heavily on pain medication have consistently been associated with unfavorable outcomes in individuals with CLBP, including increased disability and sick leave (23–25).

In contrast, engaging in active coping mechanisms that involve taking proactive steps to address pain is considered beneficial and has been linked to improved CLBP outcomes (15, 20). Other studies have identified coping strategies labelled as active, such as keeping busy or engaging in activities, diverting attention away from the pain, and engaging in physical activities or physiotherapy. These strategies were found to have no impact on the likelihood of developing a new episode of low back pain. Furthermore, having a belief in one's ability to manage pain, avoiding catastrophic thinking, and not perceiving oneself as severely disabled have been shown to enhance functional outcomes and act as mediators in the relationship between pain intensity and adjustment (17, 20, 23, 26, 27). During the anticipation of pain, individuals who utilized coping self-statements to reassure themselves of their ability to manage the pain, regardless of its intensity, exhibited lower skin conductance levels and a wider range of motion (28). Conversely, those who engaged in praying or hoping reported higher levels of pain and performed fewer activity repetitions (15). Interestingly, coping self-statements, which have been categorized as denial of pain in previous research, did not yield positive results (29, 30). Moreover, diverting attention was found to be linked to an increase in pain intensity, while feelings of helplessness were associated with depression and functional impairment in a separate study (21).

Therefore, it seems that the interpretation of active and passive coping strategies may vary across cultures (31). In addition, it is plausible that coping strategies such as distraction, praying, hoping, ignoring pain sensations, and coping self-statements may not significantly impact pain and CLBP disability after accounting for catastrophic thinking and pain self-efficacy (13). Consequently, the significance of different coping strategies could differ depending on cultural contexts and the method of assessment.

The existing evidence on the role of coping strategies in CLBP predominantly originates from high-income countries. Research on coping strategies for CLBP in African populations is limited. Previous quantitative studies suggest that existing outcome measures for coping strategies may have limited relevance in African contexts. This is because many coping strategies utilized in African countries may differ from those in high-income countries and may not be adequately represented in coping strategy outcome tools that are mostly developed in high-income countries (7, 32). This highlights the importance of first using a qualitative research approach to understand CLBP coping strategies in African populations which can then inform the development of culturally appropriate assessment tools and interventions in this context where the burden of CLBP is most significant.

Therefore, this study aimed to identify adaptive and maladaptive coping strategies utilized for CLBP through an in-depth secondary qualitative analysis of 62 interview transcripts of diverse adults in rural Nigeria and peri-urban Zambia. This study is reported according to the Standards for Reporting Qualitative Research (SRQR), a synthesis of recommendations (33).

## 2 Methods

### 2.1 Study design

The study employed a secondary qualitative research approach by analyzing previously collected in-depth semi-structured face-to-face individual interviews. The primary qualitative data used in this research were obtained from completed studies conducted in rural Nigeria (5) and peri-urban Zambia (34–36).

### 2.2 Justification for using a qualitative research approach

Quantitative research evidence from high-income countries suggests that different coping strategies may be adaptive or maladaptive in different settings and contexts (13, 15, 16–31). Current quantitative coping strategy outcome measures were developed in high-income countries and may have limited relevance and applicability in Africa. For instance, our previous quantitative study found that only 14 out of the 42 items of a coping strategy questionnaire were relevant in Nigeria. Moreover, none of these coping strategies were adaptive in Nigeria, including the ones previously identified as adaptive in high-income countries (32). These underscore the importance of an exploratory qualitative research approach that identifies adaptive and maladaptive coping strategies and their patterns of utilization and potential impact in Africa. The findings can then inform the development of relevant coping strategy outcome measures for future quantitative studies in this population.

### 2.3 Qualitative approach, research paradigm, and theoretical underpinning of this secondary qualitative research

This secondary qualitative study is based on the integration of Leventhal's self-regulatory model of illness cognitions (37–39) and Lazarus and Folkman's transactional theory of stress and coping (40–42). According to the self-regulatory model of illness cognitions, individuals perceive a potential illness through symptoms and social messages, which are then given meaning in the form of specific beliefs or illness cognitions across various domains. The concept of “Identity” refers to how individuals understand and label the illness and its associated symptoms. “Cause” pertains to personal beliefs regarding the cause of the illness. “Timeline” reflects the individual's perception of how long the illness will last. “Control/cure” assesses the individual's belief in their ability to control or recover from the illness, with subcategories such as “Treatment control” and “Personal control.” “Consequences” involve the individual's beliefs about the impact of the illness on their life. “Illness coherence” refers to the individual's sense of understanding the illness. “Emotional representation” indicates the emotional impact of the illness on the individual. An individual's illness cognitions play a crucial role in determining coping strategies, which are evaluated based on perceived effectiveness. Effective strategies are maintained, while ineffective ones lead to seeking alternative strategies (37–39, 43).

The transactional theory of stress and coping states that stress results from the interaction between an individual and their environment (40–42, 44). The present study employed a state-oriented approach to coping, as opposed to a trait-oriented approach. State-oriented coping emphasizes the specific coping strategies utilized by individuals and the associations between these strategies and various outcomes of interest (44). These outcomes may encompass coping effectiveness, emotional responses accompanying specific coping endeavors, or variables indicating adaptive outcomes such as health status or performance in a particular activity. By concentrating on state-oriented coping, this study's findings hold significance as a basis for future research endeavors focused on devising appropriate outcome measures for coping strategies and interventions aimed at enhancing coping effectiveness (44).

This study delves into the cognitive appraisal of CLBP as a threat to the individual, the individual's assessment of their coping potential, the coping strategies employed to minimize the impact on significant personal goals, the evaluation of the effectiveness of these coping strategies, and the individual's outlook on their coping potential and ability to implement additional coping strategies to achieve personal goals (37–44). Coping strategies in this study encompass both cognitive and behavioral aspects. Adaptive coping strategies are characterized by a reduced cognitive appraisal of CLBP as a threat, a positive evaluation of the individual's current coping potential, recognition of the effectiveness of the coping strategies utilized, and optimistic future expectations regarding coping potential and the efficacy of future coping strategies in achieving personal goals. Conversely, maladaptive coping strategies are defined as those linked to an increased cognitive appraisal of CLBP as a threat, a perception of limited or inadequate coping potential, ineffective coping strategies, and pessimistic future expectations regarding coping potential and the ability to implement further coping strategies to achieve personal goals. These informed the coding of the data.

### 2.4 Researchers’ backgrounds

All the authors possess extensive experience as clinicians in diverse healthcare settings, including teaching hospitals. In addition, the first and second authors are academic physiotherapists who have accumulated several years of experience in researching low back pain. Their previous published work focused on examining coping patterns among individuals with back pain in Nigeria and Zambia.

### 2.5 Study context

This secondary research is centered on qualitative data collected from 62 adults residing in rural communities in Enugu State, located in southeastern Nigeria, as well as peri-urban communities in Lusaka, Zambia. Nigeria, a West African nation, boasts the largest population in Africa, currently exceeding 200 million individuals. Furthermore, Nigeria ranks as the seventh most populous country globally, consisting of 36 states and 1 federal capital territory (45). Enugu State is among the 36 states of Nigeria, housing a population of over 3 million people, with 17 local government areas (LGAs), 14 of which are classified as rural (46). Zambia, on the other hand, is a southern African country with a present population surpassing 20 million people (47). Lusaka, the capital and largest city of Zambia, is home to over 3 million individuals. Lusaka city corresponds to Lusaka district and serves as the capital of Lusaka Province, which happens to be the smallest yet most densely populated province in Zambia. Overall, Zambia comprises a total of 10 provinces and 72 districts (47).

### 2.6 Sampling strategy and participant recruitment

The Nigerian and Zambian participants were purposefully chosen to represent a variety of sociodemographic characteristics. The 30 Nigerian participants consisted of rural adults with CLBP, selected based on age, gender, and occupation to encompass a range of situations. The 32 Zambian participants were adult nurses selected based on gender, age, workplace, educational background, area of specialization, weekly work hours, and years of nursing experience to encompass diverse experiences.

#### 2.6.1 Inclusion criteria

• People aged 18 years and above with non-specific CLBP that has lasted for more than 12 weeks. Non-specific CLBP was defined as persistent pain or functional discomfort between the twelfth rib and gluteal cleft, with or without radiation to the legs lasting longer than 12 weeks without a specific underlying pathology (43).
• People who responded in the negative to a simple “red flag” checklist with screening questions to rule out malignancy, spinal fracture, infection, cauda equina syndrome, spinal stenosis, or musculoskeletal deformity (48–50). For the Nigerian participants, this screening tool also contained body charts on which participants identified their areas of pain, the visual analog scale (VAS), on which participants indicated the levels of their pain intensity and a question about their co-morbid conditions. The tool was interviewer-administered for participants with limited literacy. For the Zambian participants, the tool asked about the duration of back pain experience and the number of sick leave days due to back pain.
• People who were residents in the selected rural communities in Enugu State, Nigeria, or who were practicing nurses in Lusaka, Zambia.

#### 2.6.2 Exclusion criteria

• Pregnant people with CLBP as CLBP due to pregnancy are not regarded as non-specific (51).
• People with any of the “red flags” listed above.
• People with cognitive impairments that limit their ability to give informed consent or respond to interview questions.

### 2.7 Data collection

This study used transcripts from previous interviews conducted in Nigeria (5) and Zambia (35) to conduct a secondary qualitative data analysis. Sociodemographic data were collected with a custom data collection form prior to interviews in the primary studies. Participants were motivated to engage actively, using prompts and probes to capture subtleties. The interviews were audio recorded and transcribed verbatim, and data validation and data saturation were confirmed in both primary studies. The primary qualitative studies explored back pain beliefs, experiences of living with back pain, the coping strategies adopted and the evaluation of these, and treatment expectations in rural Nigeria and peri-urban Zambia. In contrast, this secondary qualitative analysis provides an in-depth analysis of coping strategies by investigating different adaptive and maladaptive coping strategies and comparing their utilization in rural Nigeria and peri-urban Zambia, investigating whether adaptive and maladaptive coping strategies are used in isolation or in combination and the differential potential impact of these. Consequently, the majority of the coping strategies with their patterns of utilization and potential impact characterized in this study were not identified in the primary qualitative studies (5, 34–36). For this secondary study, only data related to coping as previously described were extracted from the primary data. The first and second authors extracted coping data from the Nigerian and Zambian samples, respectively, which were copied into a shared Microsoft Excel spreadsheet.

### 2.8 Thematic secondary analysis using the framework approach

The lead author reviewed the extracted data in the shared Microsoft Excel spreadsheet and then moved the data into NVivo version 12 for analysis. Subsequently, line-by-line coding of the qualitative data was done using a combination of deductive and inductive coding by the lead author and cross-checked by the second author. Deductive coding was guided by Leventhal's self-regulatory model of illness cognitions (37–39) and Lazarus and Folkman's transactional theory of stress and coping (40–42) as previously described. Inductive coding was utilized to identify new concepts not covered by the aforementioned theories. The data categorized under deductive and inductive codes were then used to develop analytical categories through an inductive process, which were further organized into initial thematic frameworks. The review and abstraction of initial thematic frameworks identified definitive themes, subthemes, and the narrative linking them. These analytical stages and findings were validated by the second and third authors.

## 3 Results

### 3.1 Sociodemographic characteristics of the participants

Table 1 shows the sociodemographic characteristics of the participants from rural Nigeria and peri-urban Lusaka, Zambia, in sub-Saharan Africa. In rural Nigeria, the participants were peasant farmers, some working full-time while others had part-time farming jobs alongside other jobs such as welding. In Zambia, the participants were nurse practitioners at a public health facility, providing healthcare services and community-level public health initiatives that target common illnesses.

**Table 1:** Sociodemographic characteristics of the participants from Nigeria and Zambia (*N* = 62).

Variable	Nigeria		Zambia	
	Frequency (%)	Median (IQR)	Frequency (%)	Median (IQR)
Age (years)				
20–29			6 (18.7)	
30–39	8 (26.7)		7 (21.9)	
40–49	11 (36.7)		7 (21.9)	
50–59	4 (13.3)		12 (37.5)	
60–69	7 (23.3)			
Sex				
Male	15 (50)		10 (31.2)	
Female	15 (50)		22 (68.8)	
Main occupation				
Nurses			32 (100)	
Manual labor workers	14 (46.7)			
Traders/non-manual labor workers	10 (33.3)			
Civil servants/retired civil servants	6 (20)			
Religion (Christian denomination)				
Protestant/Pentecostal	18 (60)			
Catholic	8 (26.7)			
Methodist/Anglican	4 (13.3)			
Marital status				
Married	26 (86.7)			
Widowed	3 (10)			
Single	1 (3.3)			
Educational level completed				
Primary	11 (36.7)			
Tertiary	7 (23.3)		32 (100)	
Secondary	6 (20)			
None	6 (20)			
Literacy (ability to read and write)				
Illiterate (inability to read and write)	13 (43.3)			
English	9 (30.0)		32 (100)	
English and Igbo	8 (26.7)			
Co-morbid conditions				
Hypertension/diabetes	5 (16.6)			
Knee osteoarthritis	1 (3.3)			
Pain intensity		5 (4.0–7.0)		
History of back pain experiences in months				12 (2.0–24.0)
Sick leave days due to back pain				3 (1.0–8.0)
Number of work hours per week				
<40	6 (20.0)		2 (12.5)	
40	8 (26.7)		21 (65.6)	
>40	16 (53.3)		7 (21.9)	
Years of professional experience				
5–10			13 (40.7)	
11–20			5 (15.6)	
21–30			9 (28.1)	
31–40			5 (15.6)	

SD, standard deviation; IQR, interquartile range.

### 3.2 Analytical themes

#### 3.2.1 Adaptive coping strategies

Table 2, along with the subsequent narrative provided, demonstrates the various themes and subthemes that depict the adaptive coping mechanisms employed by the individuals. These include a cognitive appraisal of CLBP as non-threatening; an ongoing perceived ability to defeat CLBP; biomedical, biomechanical, alternative, and psychosocial coping strategies that were perceived as effective; and an expectation of a future improvement in coping outcome.

**Table 2:** Adaptive coping strategies.

Themes	Cognitive appraisal of CLBP as non-threatening	Perceived ongoing ability to defeat CLBP	Biomedical, biomechanical, alternative, and psychosocial coping strategies are perceived as effective	Expectation of a future improvement in coping outcomes
Subthemes	CLBP is viewed as a normal part of a normal aging process	Perceived ability to continually engage in exercise and sporting activities as a means of defeating CLBP	Lying on hard surfaces with or without manual techniques temporarily relieves symptoms	Perceived inherent ability to continually defeat back pain which is expected to lead to an improvement in the coping outcome in the future
	CLBP is regarded as episodic and only related to engagement in hard manual labor	Perceived ability to use analgesic drugs obtained over the counter intermittently to temporarily defeat episodic CLBP	Avoidance of heavy lifting and sustained heavy manual work, plus activity pacing provide long-lasting pain relief but are difficult to sustain due to competing needs	Planned changes in conventional healthcare
		Perceived ability to continually pace activities to defeat CLBP	Avoidance of sustained static or awkward postures with or without intermittent stretching provides pain relief but this is difficult to sustain due to competing needs	Planned changes in personal lifestyle choices
			Engagement in exercise as a coping mechanism	Expectation of God’s intervention that will improve coping outcomes in the future
			Promotion of inner peace	
			Distraction strategies for minimizing pain perception	
			Use of cold therapy alone or in combination with other strategies	

##### 3.2.1.1 Cognitive appraisal of CLBP as non-threatening

Most of the participants regarded CLBP as a normal part of the aging process and as episodic and only related to engagement in hard manual labor. Several participants held a non-threatening perspective toward CLBP for several reasons. As such, these individuals opted for diverse home remedies and refrained from seeking conventional medical assistance in a formal healthcare environment. Furthermore, some participants believed that experiencing CLBP was a normal occurrence and not a sign of a severe medical condition. This was because they did not suffer from CLBP in their youth but only began to experience it as they aged. In addition, some participants did not view CLBP as a cause for worry as they perceived their pain to only occur during times of strenuous physical exertion and would disappear once they stopped engaging in such activities. They believed that their physically demanding jobs and duties or other daily tasks were associated with their experiences of back pain, particularly because the pain was intermittent rather than constant. Consequently, this intermittent nature of the pain provided them with a sense of reassurance.

“I take it that it [back pain] is a natural thing or because I am getting older … when someone was a young man, the person may be able to jump, do anything without having any problem, bible explains this. But as long as you are getting older, anything you do, you will get some reaction and that reaction, you won’t take it that something very bad has happened. It happened at its time. So, I don’t agree that it is a serious ill-health … it's due to the fact that we are getting older … ” (Nigerian Patient 10).

“For me I think it is just age … I think when I reached around the 40s the problem started. Every time after knocking off I make sure I do a bit of jumping and jumping because if I don’t that day, I will have terrible back pain …. and the following day it would be horrible, but I think age has also contributed otherwise it is not very bad I’m managing.” (Zambian Nurse 3).

“ … I have never seen myself as someone having back pain … Sometimes during lovemaking, there is a way you would push yourself to show ability as a human being, sometimes you feel the pain. But after a little while it stops …  I have never gone to the hospital because of it [back pain]. It has never given me great worries or given serious thoughts to it, whenever it starts like that, I use that therapy, it then stops … ” (Nigerian Patient 10).

“If the day is so busy, actually immediately after work I experience the back pain. But that back pain won’t finish within a day. Maybe it's due to even old age, It will take two to three days, if it's still there, unless I take a Panadol, then it subsides.” (Zambian Nurse 2).

“Sometimes with the workload there are just too many clients or patients that one is taking care of, so by the time you are knocking off work, just doing the nursing of those patients, your back will pain, yes …. I think especially in patient wards with staff shortage lifting the patients from the bed to the stretcher and stretcher to bed I find that very straining to the muscles because the equipment that we can use for lifting patients, or transferring patients, and all is not there.” (Zambian Nurse 9).

##### 3.2.1.2 Perceived ongoing ability to defeat CLBP

Several participants shared their perspectives on the ongoing challenge of managing CLBP. They cited the perceived ability to continually engage in exercise/sporting activities, the use of analgesic drugs obtained over the counter, and a continuous pacing of activities to defeat CLBP. However, they emphasized the need for expertise in handling CLBP through exercise, medication, and adjusting activity levels, as well as the mental determination to persistently strive for productivity despite the discomfort. The participants demonstrated their ability to consistently surpass the limitations imposed by CLBP in their daily lives by implementing various strategies such as the use of over-the-counter pain medication to cope with the discomfort while continuing to work. Only a few participants believed that engaging in physical activity or sports during their younger years contributed to their ability to maintain fitness as they aged. These individuals were confident that such activities helped them to manage CLBP and maintain independence. Furthermore, the majority attributed their back pain to stress and lack of rest, thus, they found relief by pacing their activities, but this was challenging in rural Nigeria.

“ … I’m not happy that I have pains … I resist it … the grace to resist it is always on me to still do my work. There's no time it has ever hindered me … Myself and it [back pain] …. a man must do his best in this world because one will not sit in one place and wait for death. You will be struggling to see if you can defeat the sickness that came. So, I try … myself and it [back pain] are really fighting … ” (Nigerian Patient 14).

“Towards the back pain that I feel I find, that as men on the ward, you are looked at as the muscle. So, any heavy lifting that comes about, you are the go-to person. So, we just disregard the pain to help lift.” (Zambian Nurse 5).

“ … I resist that. I resist that [back pain]. Then I use self-medication. Sorry to use that word. Go to chemist to take one or two drugs if it becomes tensed … but nevertheless, I am a sports man. I was involved in athletics in my school days, and it helped me a lot. Because when this thing [back pain] comes, I resist it to the letter.” (Nigerian Patient 15).

“When you have the back ache, of course what usually happens is you take a couple of paracetamols or Brufen and continue with work. When you have back pain it's not time to go and rest or lie down … every time after knocking off I make sure I do a bit of jumping because if I don’t that day I will have terrible back pain …  and the following day it would be horrible.” (Zambian Nurse 6).

“I usually take drugs, after taking the drugs, even sometimes unless I lie on the bare floor before I can be relieved.” (Nigerian Patient 10).

##### 3.2.1.3 Biomedical, biomechanical, alternative, and psychosocial coping strategies perceived as effective

Various approaches were explored by the participants to alleviate their pain. They expressed their belief in the effectiveness of biomedical, biomechanical, alternative, and psychosocial coping strategies. The participants indicated that a range of biomedical strategies, such as medications and other techniques supported by mainstream healthcare providers, were recommended. Biomechanical methods were suggested by healthcare experts within the community such as physiotherapists. Alternative therapies were proposed by notable individuals, community members, or practitioners of complementary medicine, whereas psychosocial coping mechanisms were derived from family, religious figures, or the community. For instance, many participants found that lying on hard surfaces such as the bare floor or mats during an active episode of back pain relieved their back pain whilst lying on a soft surface such as a mattress made the pain worse. When insufficient to relieve pain, some participants had to combine lying on a hard surface with manipulation strategies from family or community members.

“ … [I]f I lie on the mat without mattress. Because sometimes when I lie on the mattress, when it starts and I lie on the mattress, it gets very painful. But if I lie on the bare floor, place a mat on the floor, it makes it better. That's how I do it until it subsides … ” (Nigerian Patient 10).

“ … [F]or me to get relief I have been sleeping on the floor using a thin mattress the past 3 years … ah my body and back pains a lot whenever I sleep on the bed.” (Zambian Nurse 10).

“ … So when I come back from work, any day I come back from work and carelessly lie on this mattress, and remain there till morning, this back pain becomes worse. So when I come back from work, I usually lie on a hard surface like on the floor or on a mat, somewhere that is hard. So the next morning I will feel okay. So this is how I deal with my back pain. I try my best to keep to all these things so that it doesn’t truncate my work the next day.” (Nigerian Patient 3).

“… Sometimes after lying there, it [back] becomes very painful. It won’t stop. Then I call people, I lie flat on the floor, the person would be stepping his legs on my back … after I use balms … .to massage my muscles at my back … ” (Nigerian Patient 15).

“ … Another way is that I use shear butter, I buy it, I rub it. After massaging it, it makes it cool down, then after a while, it starts, I will say ‘right’, when it comes … there is the shear butter, when I get home, have my bath, I am massaged with it and it cools. That's it. There is nothing else … ” (Nigerian Patient 11).

The relief derived from the manipulation strategies was often linked to a sound that participants explained was from the bones of the back setting back in place. However, some other participants linked the perception of a similar sound to be associated with heavy lifting and injuring their backs. Some of these participants reported feeling more pain when this happened, and they used spiritual coping strategies to deal with the ensuing pain. In contrast, other participants said that the manipulation strategy worked even when pain medications did not, as narrated below.

“ … [B]ut when it starts, it really hurts seriously. I try to lie down, impossible, I sit down, impossible. I lie on my belly, and the children and my neighbours will be stepping on my back at that place. After stepping on it for some time, you hear the sound ‘kpum’, I can then lie properly and sleep.” (Nigerian Patient 11).

“ … [T]here are times when they are stepping on it, it makes the sound to show me that it has gone into its normal place and at that moment, the pain stops immediately … ” (Nigerian Patient 10).

“ … [W]hen I lift something in a basin like cassava, after peeling the cassava, I go to lift it up, it does like this … it sounds ‘kim’, I shout ‘Jesus’ …  I say God … My Lord will come, He answers me at that time I called him, it stops … I don’t play with church. Once I go to the church … Yes (nods). Once the Pastor has prayed for you, all your pains will go away.” (Nigerian Patient 13).

“ … Only my daughter, the only thing she does for me when it starts, I tell her to climb on my back, and match on my back. After matching on my back … sometimes it seems like it got worse ….  Sometimes it seems a bit better … ” (Nigerian Patient 8).

Several participants experienced a reduction in back pain symptoms by avoiding heavy lifting and other strenuous manual work. This relief was maintained until they resumed heavy lifting or manual work. Some participants even reported a complete disappearance of back pain symptoms when they consistently avoided heavy lifting or manual work in the long term. Consequently, they refrained from engaging in such activities for an extended period. This long-term alleviation of back pain symptoms also led to the abandonment of maladaptive coping mechanisms such as prolonged use of pain medication and dependence on analgesic drugs, including opioids. However, many participants in rural Nigeria found it challenging to avoid heavy manual work in the long term due to their informal self-employment status. They had to continue with manual labor to meet the financial needs of their families. In contrast, some participants opted to moderate their daily involvement in manual work instead of completely abstaining from it as a strategy to manage their back pain.

“ … What makes it [back pain] better is if I don’t lift anything, it won’t disturb me. So I lifted the engine, after lifting the engine, this back pain started hurting me. Up till now, once I lift something that is heavy, it hurts me … ” (Nigerian Patient 12).

“ … [W]ith staff shortage I feel like lifting the patients from the bed to the stretcher and stretcher to bed is so straining on muscles of the back.” (Zambian Nurse 9).

“..[I]f I don’t do cement job, or farm work and just stay on my own, then the pain is gone … Any time I stop moulding blocks. For example, last 3 weeks, for almost one month and 3 weeks, I didn’t go to work and I was okay. So I started work yesterday, and it [back pain] started … But when I stop work, like when there is no job, I stop taking these daily drugs … ” (Nigerian Patient 3).

“During night duty, especially in labour ward, we are working for so many hours, no time to rest not even a bit. You start at 6:00 in the evening and you are standing the whole night up to 6:00 in the morning, by the time it's morning, ‘eish’, your back is paining … but when you’re on nights you are just okay no pain.” (Zambian Nurse 10).

“ … Sometimes when it [back pain] happens, I bear it, when I stay in one place, it feels cool and then I can continue whatever I am doing … I don’t do the farm work all the time, I do the much work I can do, come back home, and eat, have my bath, then rest … ” (Nigerian Patient 1).

Many individuals noted an increase in back discomfort following prolonged periods of static body positions but found relief after changing positions. While some of these individuals did not attribute their back pain to strenuous manual labor, others reported that both static postures and manual labor exacerbated their pain. Engaging in stretching and movement after being in static positions helped alleviate their pain. Unfortunately, some participants felt compelled to continue in these aggravating postures due to their dependence on them for their livelihood.

“… [I]f I bend now, and stay long in that position, it hurts me slightly. When I stand straight and do a little exercise, then I become okay … What makes it better is if I am moving my body about, it gets slightly better. But when I sit down, it gets worse, if I try to get up, it's so difficult …” (Nigerian Patient 5).

“ … We usually conduct a diverted outreach and we palpate mothers on the ground, so you have to kneel and bend … you go up and about, bending, doing all sorts of things, … then we have a lot of mothers that we are supposed to palpate, so by the end of palpation you have back pain and it lasts for more than three days.” (Zambian Nurse 22).

“Like the way I am sitting down now, I would say I am comfortable the way I am sitting relaxed. If I get up now, I won’t have pain … If my legs are awkwardly positioned, this shows that I am not sitting very well. So after a little while … I am unable to spread my legs. So sitting for a long time will do that … hence if I sit for a little while, I stand up, and do a little exercise …” (Nigerian Patient 6).

“ … The other thing is when we are suturing, we need to sit on a comfortable chair but, we are standing and bending most of the time, the moment you finish you can’t even stand up right you can have backache even two or three days … Even standing, because we stand a lot, so you find that the time you want to sit you feel the back as if something has been … oh, your back has been locked, yes … ” (Zambian Nurse 21).

“ … I’m a furniture maker. So, I may lift wood, I may lift something that is heavy, I may be sitting on a chair, there is a time you will get to, and you want to get up, you will groan ‘mmm’ … Groaning like that means that the pain is holding you. So, after a while you stand straight and stretch your back, walk around a little and it [back pain] stops … ” (Nigerian Patient 8).

“As of me … , it's like more hours of sitting, because the department I am in, the moment I report after setting the trolley my work is to sit. I find myself sitting for up to maybe the end of the activity, maybe late afternoon or so. So, by the time you stand, you feel, you experience the back pain … ” (Zambian Nurse 4).

“I don’t do hard labour to say that it is the hard labour that caused my back pain. When I am idle like I am now, before I am able to sit up … but if I am moving my body around, yes … I feel better … I just manage it. Even where I do my trading, the thing disturbs me, where I sit to do my trading. It disturbs me but I manage it because without that you cannot feed well … ” (Nigerian Patient 5).

Some people exercised to cope with back pain, which reduced the severity but did not eliminate it completely. A few participants used spiritual practices, religious rituals, and social support to find inner peace and manage the challenges of chronic pain. These strategies improved sleep quality and allowed them to engage in meaningful activities. Spirituality helped them cope with pain, stress, anger, and frustration by promoting forgiveness and hope in a higher power. Spiritual practices also helped them relax and sleep better, reducing the impact of back pain on their daily lives. Religious activities gave them hope and faith in resolving their chronic pain. Overall, spirituality helped some participants adapt and thrive despite ongoing pain. A small number of participants used work-related tasks and religious practices as distractions from their back pain.

“ … [S]ince I started exercise, it is reducing … I do the exercises … the thing [back pain] was reducing, small, small … but still it is not completely gone … the day before yesterday, I was jogging, sweating … exercising yourself helps a little … ” (Nigerian Patient 14).

“I noticed if I don’t do exercise I wake up in the morning and I come to work I won’t even be able to work. So, I do the exercises that I was taught every day and I have to put on belts. So, these days at least I am able to move around … ” (Zambian Nurse 6).

“ … What the bible says concerning that is that you should follow everyone with peace, there was no one it said should be excluded. Another is that you should have forgiveness for people like this. That you shouldn’t allow evil to overcome you rather use good to overcome evil. Romans chapter 12 is where you will see this. Place your hope in God. Place all your troubles on Him. Have faith that it will be well … ” Nigerian Patient 6).

“If it were not for all the prayers I go for, I don’t know if I will be a living person today. Thank God in all my situation, I thank Him, He knows everything. There was one woman that does prayers in our neighbouring village, I was taken there. She is a prophetess … she was using prayers to destroy all those things, there was also something she rubs on me … ” (Nigerian Patient 2).

“ … [L]ike work that I’m not able to do. I call her [wife] and explain to her the problem that I’m having, like some work, like the ones she can do. She helps me to do it. My mother also assists me. Another help is this way my child came back, if there is anything I want to do, he helps me to do it. It is no longer me doing things by myself … ” (Nigerian Patient 10).

“ … Like the baluster we put in the church, I was the one that did it … the tile … in my house … I did also … when you do it and come to see it, you will be happy … Yes … when I’m doing things like this … bible says in the book of proverbs, 17 verse 22 that gladness is medicinal … joy that is happiness. When I start to do things … when I’m doing the things that I’m called for, I am always happy … and they are antidote to a lot of things that run against my health … ” (Nigerian Patient 15).

Several participants mentioned utilizing various forms of cold therapies, such as taking a cold bath in the morning at a nearby stream or at home, in order to alleviate the severity of their back pain. However, none of the participants experienced complete resolution of their back pain. Consequently, a small number of them expressed hopes for a more favorable outcome in terms of alleviating their pain symptoms, either through their existing coping strategies or by adopting new methods in the future. Only a handful of participants believed that their current coping strategies were effective and adequate enough to continue diminishing the impact of their back pain on their daily lives.

“ … [A]fter troubling me for some time … I go and immerse my waist  …. very early in the morning by 5 o’clock, I go and immerse it inside cold water at the stream … It helps me … ” (Nigerian Patient 9).

“Pain relief is not always drugs it can be cold compress, hot compress sometimes massage, back rub.” (Zambian Nurse 11).

“ … [T]he best thing is that you get ordinary water in a bowl and keep it outside, then you leave it outside till morning, by then the dew must have fallen into it, to make it very cold … Then, if I keep doing it like that, and try to lie on the bare floor and not on the mattress … ” (Nigerian Patient 10).

##### 3.2.1.4 Expectation of a future improvement in coping outcome

Several participants expressed a desire to enhance their involvement in traditional healthcare to enhance the efficacy of their coping mechanisms and alleviate the effects of their back pain. A small number of participants also sought to make additional lifestyle adjustments to minimize stress and its influence on their back pain. Other participants held the belief that divine intervention would lead to healing in the future. Specifically, they anticipated that reduced physical labor and an improved quality of life, facilitated by God's intervention in their children's income potential would alleviate the severity and impact of their back pain, ultimately enhancing their coping strategies.

“ … So I want to adjust my time and my timetable and my programs. So that's the thing. Presently I had a serious … there was a time that my stress was too much … I also changed … started taking fruits more … ” (Nigerian Patient 15).

“And also stress management sometimes you know we are stressed maybe financial stress, and we are very good at caring for the patients, but for ourselves it's not there. So, we find that you are stressed literally the whole body is aching, but we are not able to manage our bodies … ” (Zambian Nurse 1).

“ … If it wasn’t for God's intervention or God's healing in short … I wouldn’t be here, it's [back pain] not something I came to life with, if it was someone that did it to me, I know that one day, God will surely heal me, God will surely provide means to treat it, I wasn’t born with it … ” (Nigerian Patient 2).

“ … [W]e also need to watch our diet because of obesity. Obesity is really an enemy to health … it contributes to back pain, so we need maybe also to take more care … ” (Zambian Nurse 3).

“ … If God blesses me and blesses my children, then I will sit down and they will be bringing me everything and I will be eating, I will just relax, and stop all this hard labour, hard labour, hard labour … ” (Nigerian Patient 1).

#### 3.2.2 Maladaptive coping strategies

Table 3 and the subsequent narrative below illustrate the themes and subthemes representing the maladaptive coping strategies utilized by the participants. These include a cognitive appraisal of CLBP as threatening, the impact of biomedical, biomechanical, and alternative management strategies being transient and viewed as ineffective, and the perceived inability to improve coping outcomes with catastrophic expectations for the future.

**Table 3:** Maladaptive coping strategies.

Themes	Cognitive appraisal of CLBP as threatening	Impact of biomedical, biomechanical, and alternative management strategies is transient and viewed as ineffective	Perceived inability to improve coping outcomes with catastrophic expectations for the future
Subthemes	Perceived inability to control the impact of CLBP on livelihood	Dependence on over-the-counter analgesic drugs for daily function	Unavailable social support to improve coping outcomes
	Inability to understand the cause of CLBP	Conventional biomechanical and behavioral strategies did not eliminate pain and were perceived to be ineffective	Perception that the elimination of pain would warrant giving up the source of livelihood in the absence of mechanization to reduce manual labor
	Understanding of CLBP as an illness		Perceived inability to afford a conventional treatment that is believed to eliminate pain

##### 3.2.2.1 Cognitive appraisal of CLBP as threatening

Several participants perceived CLBP as a threat because they believed they had no control over its negative effects on their work and overall wellbeing, struggled to identify its underlying cause, considered it a disease, and were aware of the persistent nature of the pain. Those who felt powerless to mitigate the impact of CLBP on their physically demanding occupations felt threatened by the condition. The prevailing notion that back pain was linked to strenuous labor and hardship contributed to this perception. Consequently, individuals who did not engage in manual labor did not see themselves as leading a life of hardship. They believed that they were socioeconomically secure, yet experienced CLBP. Consequently, they were puzzled by the presence of back pain. As a result, they viewed CLBP as a threat. However, the majority of participants understood CLBP as a condition that hinders the achievement of one's original life goals. Others viewed the presence of long-lasting pain as spreading to other areas of the body. They perceived CLBP as an illness that prevented them from fulfilling their expected social roles and engaging in daily activities. Consequently, due to this perception of CLBP as an illness and the persistent nature of their back pain, certain participants held onto the hope of an immediate and permanent recovery from their pain. In addition, some Nigerian participants believed that the cause of CLBP was the unfavorable living conditions prevalent in rural areas, characterized by constant suffering and the physically demanding nature of their occupations.

“ … Welding, fabrication. You know our job implies carrying heavy things. So when I remember it, when the thing [back pain] starts troubling me, and I remember that I have to lift something, like moving my welding machines around and other things I use to do this work. It gives me problems. I feel down whenever I remember the thing.” (Nigerian Patient 10).

“ … God blessed me with children. Is it material things that I don’t get? It's just that inside my body, I don’t get myself. It's not worry that I don’t get this or that that is my problem …. It's just that we don’t understand how ill health comes to someone … ” (Nigerian Patient 5).

“ … Ill health is obstacle … because it makes you … if you had focus, when you remember that there is an illness in your body, that may prevent you from … the height you want to reach, it makes someone's spirit to go down …. And that is what happens to me … ” (Nigerian Patient 10).

“ … Back pain is illness, I see myself as someone with ill health …. I have ill health, because I’m not sound within me, but people looking at me will think I’m sound …. Like someone with headache, someone with arm pain, someone with back pain, someone with stomach pains, that is the person that has ill health. And I am suffering from one of these things because I have back pain …  ” (Nigerian Patient 12).

“ … [W]hen your mates are drinking, you won’t drink saying that you are not healthy. When your mates are going to the farm, you won’t go, when your mates are running, you won’t be able to run like your mates because you are not healthy … If I see something that will heal me of this, make it heal immediately, without coming back, it will be better for me. Because there is no one who will be happy with illness in their body … ” (Nigerian Patient 1).

##### 3.2.2.2 The impact of biomedical, biomechanical, and alternative management strategies is transient and viewed as ineffective

Participants used a range of healthcare strategies, including both traditional and alternative approaches, to manage their back pain. These strategies provided temporary relief or long-term reduction in pain but did not completely eliminate it. Many participants turned to over-the-counter drugs or herbal remedies but found them ineffective due to their short-lived effects and lack of professional guidance. Some participants became reliant on these substances to carry out their daily activities. A few sought treatments from conventional healthcare practitioners. Although these strategies offered some long-term relief, they did not eliminate the pain entirely.

“ … I have tried native medicine …. They are just the ones sold in the market, I buy them, to try to see if it will get better. After trying it, it happened that it was useless. What was happening continued. Since that time, I didn’t go to buy drugs any longer, and the ones that were given to me at the chemist. I have tried all of them, there was no improvement. So I left them since then … ” (Nigerian Patient 7).

“ … It is getting to 3 years. If I try to take drugs, it stops briefly, after a little while, it starts again … even the computer people came to our place the other day … I complained about my back pain, they gave me drugs … I bought those drugs, took them, still the back pain continued … ” (Nigerian Patient 5).

“When experiencing the back pain and you have to work …. that back pain won’t finish within a day unless you take a Panadol. And because now we are aging Panadol does not even work so, we take something heavier ….you go to an extent of taking Diclofenac … ” (Zambian Nurse 8).

“ … [T]here is no drug that I have not taken, like all these food supplements and herbal remedies like ‘yoyo peters’, like all these drugs they sell everywhere, there is none that I have not bought at least to wash your system and yet … it will subside briefly, once I stop taking the drugs, it continues from where it stopped … I am tired, there is no drug that I have not bought even the drugs that are sold in public transport buses. When I start the drugs, the pain will behave like it is shifting a little, once I stop it, the pain starts. Therefore, I don’t stay without drinking analgesics … ” (Nigerian Patient 2).

“ … After a while, I started going to XX [teaching hospital], they took X-rays, after that, one of my doctors who was working there, sent me to Orthopaedic hospital, he took X-ray, examined me, and started giving me treatment. Gave me the treatments for a while and sent me to physiotherapy. So, I was having the physiotherapy, then went to … other things … I was being massaged … I was also given some exercises, I do the exercises … small, the thing [back pain] was reducing, small, small … but still it [back pain] is not completely gone … ” (Nigerian Patient 14).

“I was told to be sleeping. I was told to not lie flat, not to lie on my belly. Just sideways like this. Sometimes I stay like this on the mattress (demonstrates it) … but the back pain is reducing but has not gone.” (Nigerian Patient 14).

##### 3.2.2.3 Perceived inability to improve coping outcomes with catastrophic expectations for the future

Many participants harbored pessimistic expectations regarding the future and doubts about their ability to cope with their back pain. A portion of these individuals perceived a lack of support from their community, significant others, and family members in effectively managing their pain. A few participants believed that eliminating their back pain would necessitate relinquishing their manual labor jobs, which served as their primary source of income. They perceived the government as being indifferent to their struggles and unwilling to invest in mechanization to alleviate their exposure to physically demanding work. Conversely, some participants held the belief that traditional treatments could alleviate their back pain, but financial constraints prevented them from affording such treatments. Many Nigerian participants aspired to secure lighter job roles that involved fewer manual tasks, yet they perceived this goal as unattainable for themselves.

“ … But if it is now that it is caused by the kind of job I do. Unless I am told to stop this job, and given another job to do that is lighter, back pain will go. That is just the simple logic. Leave this job and back pain stops. Continue this job, then you and the back pain will continue … ” (Nigerian Patient 3).

“ … What would help me is if there is solution that the government will bring out for the thing, a way that the government will bring out to help us … we don’t have those machines that lift engines … because … our country is very poor, we don’t have all these things … ” (Nigerian Patient 12).

“ … There is something that is called special treatment, it [back pain] will heal, if I receive special treatment … But I haven’t had money to treat it. I haven’t had money to go to a good hospital … If you go to a good hospital that is not all these rubbish hospitals, they will give a huge bill, but you will be treated … ” (Nigerian Patient 4).

“ … If I go to a good medical doctor, people that are well educated, that are specialised in this my illness that is troubling me … If I can afford it … and they check my body very well, and tell me what to be doing, and tell me the drugs to take, tell me what to do so that my illness goes completely … ” (Nigerian Patient 10).

## 4 Discussion

This secondary qualitative study investigated the adaptive and maladaptive coping strategies utilized by a diverse group of adults with CLBP in rural Nigeria and peri-urban Zambia. The participants employed a range of coping strategies, influenced in part by their perceived effectiveness in alleviating back pain.

A key finding was that most individuals employed a combination of adaptive and maladaptive coping strategies to manage their pain which aligns with research evidence suggesting that people use both adaptive and maladaptive strategies simultaneously to manage chronic pain (52). In addition, the study revealed that many coping strategies for CLBP were common among these very diverse sub-Saharan African populations, regardless of educational background, socioeconomic status, or rural/urban residence. This may be due to the similarity of the two sub-Saharan African cultures. Culture influences coping strategies as well as neurophysiological processing of nociceptive information and psychological, behavioral, and verbal responses to pain through supraspinal influences on pain perception (53).

The adaptive coping strategies frequently used by the Nigerian and Zambian participants included maintaining a non-threatening perspective on CLBP, having positive beliefs in their ability to minimize its impact on daily life, and utilizing various biomedical, biomechanical, and psychosocial coping techniques such as lying on hard surfaces, avoiding prolonged manual labor and static postures, engaging in physical exercise, applying cold therapy, using manual therapy, and making lifestyle changes to mitigate the effects of CLBP in the future. These findings align with research evidence showing correlations between the clinical outcomes of back pain and the firmness of sleeping surfaces, the perception of CLBP as a threat, engaging in heavy manual work, maintaining static postures, engaging in exercise, utilizing cryotherapy, and receiving manual therapies (7, 54–62).

Among both Nigerian and Zambian participants, it was observed that a common maladaptive coping strategy was relying on analgesic medications for daily functioning. However, it is important to note that the use of analgesic medications has limited effectiveness in managing CLBP and is associated with adverse effects on the cardiovascular, gastrointestinal, and central nervous systems (63–65).

Nevertheless, there were subtle differences in the coping strategies employed by the Nigerian and Zambian participants. Research evidence suggests that rural residency and low socioeconomic status are associated with an increased likelihood of chronic pain but it is unclear how these factors influence coping strategies (66). Notably, people of lower socioeconomic status and education tend to have less control and less ability to cope with their pain (67). Unlike the Zambian participants, the rural Nigerian participants, who were mostly of lower socioeconomic status, utilized both adaptive and maladaptive spiritual coping strategies. These included relying on spirituality to promote inner peace and maladaptive spiritual coping strategies, such as expecting spiritual healing. It is worth noting that the Zambian participants did not report utilizing spiritual coping strategies. This may be attributed to the professional setting of the interviews conducted within hospitals where they were employed, in contrast to the Nigerian participants who were interviewed in their homes. Differences may also be because of the lower socioeconomic status of participants in rural Nigeria which has been linked to a greater likelihood of using spiritual coping. It has been suggested that people in disadvantaged circumstances compensate for their situation and obtain rewards that would otherwise be out of reach through religion which may reduce the negative psychological effects of adversity in daily life (68, 69). In addition, the Nigerian participants employed other adaptive coping strategies that were not reported by the Zambian participants, including pacing their manual activities, utilizing distraction strategies, and making planned changes in their conventional healthcare approaches. These differences in coping strategies could potentially be attributed to the challenging living conditions experienced by people of low socioeconomic status in rural Nigeria, which may differ from the nurses in the peri-urban settings in Zambia.

The effectiveness of adaptive pain coping strategies varied among individuals, as what worked for some participants did not work for others, and *vice versa*. This discrepancy in effectiveness may explain why previous research found that none of the coping strategies in the Coping Strategies Questionnaire were adaptive in Nigeria (32). In addition, all coping strategies, including those considered adaptive in Western cultures, were positively associated with self-reported disability and pain intensity (32). This finding suggests that a case-by-case analysis of coping strategies may better reveal the role of coping strategies in quantitative studies. It is also possible that the simultaneous use of maladaptive coping strategies may have overshadowed the benefits of adaptive coping strategies in previous quantitative studies. Therefore, rehabilitation programs should focus on identifying and implementing intervention components that promote the use of individual-specific adaptive coping strategies while discouraging the use of individual-specific maladaptive coping strategies using a case-by-case analysis.

The transition of participants from one coping strategy to another, whether adaptive or maladaptive, lacks clarity in terms of the underlying mechanism. However, based on their narratives, this transition could be linked to specific physical, psychological, or social pressures on participants at particular moments, as well as the perceived immediate success or failure of the coping strategy currently in use. These findings align with the literature that suggests that people adapt their coping strategies over time based on personal experiences, trial and error, health professional advice, and social learning through observing significant others (70, 71). Moreover, the findings indicate that the cognitive assessment of CLBP as either threatening or non-threatening, along with positive or negative expectations regarding the future effectiveness of coping strategies, seem to play a role in determining the coping strategies chosen. This discovery is consistent with prior quantitative research conducted in Nigeria, which revealed that stronger illness perceptions that indicate a more threatening perspective of back pain were the most significant predictors of both self-reported and performance-based disability (7). Taken together, these earlier findings and the outcomes of the present study strongly imply that coping strategies could be a crucial mediator for numerous CLBP clinical outcomes. Nevertheless, the impact of specific coping strategies seems to vary among individuals, and the existing tools for measuring coping strategies may not fully capture this concept and its unique characteristics.

Our results indicate that only a small number of participants seemed to utilize adaptive coping mechanisms primarily. For example, “Nigerian Patient 6” demonstrated a predominant use of adaptive coping strategies, which included maintaining a non-threatening perspective on CLBP, avoiding or addressing prolonged static postures through intermittent stretching, regularly engaging in exercises, pacing heavy manual activities, incorporating spirituality for inner peace, believing in the efficacy of his coping strategies for the future, and never seeking nor intending to seek treatment for his back pain. This amalgamation of adaptive coping strategies is a unique finding in this study. It aligns with qualitative research findings that propose a non-threatening perspective on CLBP as a key factor influencing the adoption of adaptive coping strategies in rural Nigeria, with the opposite also holding true (5). Therefore, integrating a variety of adaptive coping strategies may prove beneficial in back pain rehabilitation programs.

The present study identified the utilization of spirituality as one of the effective coping strategies. However, previous quantitative research conducted in Nigeria revealed that praying and hoping were counterproductive and linked to self-reported disability and pain intensity (7, 32). Conversely, qualitative research indicated that spirituality could be either beneficial or detrimental depending on its application within this Nigerian population (5, 6). Specifically, maladaptive aspects of spirituality were found in the form of attributing pain to spiritual causes and holding catastrophic spiritual beliefs, whereas adaptive aspects were observed in pain acceptance and social support derived from spirituality (5). The current study further demonstrated the adaptive use of spirituality in finding inner peace and fostering hope for a better future, both of which aided in coping with back pain. Consequently, the absence of differentiation between adaptive and maladaptive use of spirituality may account for the positive associations between praying, hoping, and self-reported disability and pain intensity in previous quantitative studies that employed coping strategy questionnaires (7, 32). This amalgamation of adaptive and maladaptive coping patterns may also elucidate the failure to identify adaptive coping strategies in other populations using different outcome measures (72), necessitating the utilization of structural equation modeling in studies aiming to discern distinct coping patterns associated with positive clinical outcomes (73).

Based on the accounts provided by participants in the present study, it is evident that the adaptive coping mechanisms implemented only offered temporary relief from symptoms, except for sustained avoidance of strenuous manual labor while engaging in other less physically demanding tasks. Research indicates that engaging in heavy manual labor is linked to prolonged sick leave (74, 75), continued pain (76), and job abandonment (77). Nevertheless, it remains uncertain whether there was a lasting remission of back pain symptoms in the long term, as the participants were unable to maintain the avoidance of heavy manual work due to familial obligations and financial constraints associated with their low-income self-employment status. Previous studies in affluent nations have shown that individuals who transitioned to lighter duties were more likely to experience sustained relief from mild back pain symptoms (78). However, other studies have found that once factors such as pain severity, workers’ compensation, gender, personality, depression, and injury severity are taken into account, the physical demands of a job may no longer influence one’s return-to-work status (79–81). These findings were in high-income countries with a social benefit system which does not exist in Nigeria, and so factors affecting one’s return to work may very well differ in Nigeria. Subsequent research should therefore explore the potential for sustained long-term relief from back pain symptoms and enhanced clinical outcomes among individuals in these African communities who shift from strenuous manual occupations to less physically demanding roles. However, these investigations should also consider the impact of changes in income and job stability, such as moving from temporary to permanent contracts, on the outcomes observed.

## 5 Strengths and limitations of this study

Using a secondary qualitative research design was cost-effective and time-saving, allowing access to and synthesis and comparison of a large amount of qualitative data from different countries. The authors led the primary studies on which this study was based. Therefore, there was confirmation of the quality, reliability, credibility, and trustworthiness of the primary qualitative data with validity confirmed in the primary studies. The access to complete data ensured data richness. An understanding of the context in which the original data was collected ensured the correct interpretation and application of findings. Interpretation bias was avoided because the authors of the current study led the primary studies. However, this study was not free of the limitations associated with secondary qualitative research. For instance, coping strategies were only a part of several factors that were investigated in the primary studies. Hence, there could have been less questioning of the participants in relation to the trajectories of different adaptive and maladaptive coping strategies that was a focus of the current study but was not the primary focus in the primary studies. We did not collect data on the intensity of pain and co-morbidities for the Zambian participants and the history and duration of pain for the Nigerian participants which limited the understanding of how these factors may have influenced coping strategies.

## 6 Conclusions and recommendations for practice and future research

This research has identified numerous adaptive and maladaptive coping strategies employed by adults with CLBP in rural Nigeria and peri-urban Zambia. The study revealed that individuals utilized a combination of various adaptive coping strategies as well as a mix of different maladaptive coping strategies to manage their back pain. Considering these findings, it is imperative to conduct additional research to develop outcome measures that encompass the wide spectrum of coping strategies. It is crucial for both clinicians and researchers to recognize that certain coping strategies can be employed both adaptively and maladaptively, and to acknowledge the varying impact of specific coping mechanisms on diverse individuals, communities, and societies. Moreover, further investigation is warranted to pinpoint intervention strategies that can promote the adoption of adaptive coping mechanisms while simultaneously discouraging the adoption of maladaptive coping strategies in these populations in sub-Saharan Africa. This knowledge can serve as the basis for designing rehabilitation programs aimed at mitigating the negative effects of CLBP in these populations, with potential applicability to other demographic groups.

## Data Availability

The data analyzed in this study is subject to the following licenses/restrictions: This secondary qualitative investigation relies on qualitative information obtained from previously completed research. Additional inquiries may be directed to the corresponding author due to ethical restrictions. Requests to access these datasets should be directed to the corresponding author at chinonso.chidobe@unn.edu.ng.
